# Bioactive metabolites produced by *Streptomyces Cheonanensis* VUK-A from Coringa mangrove sediments: isolation, structure elucidation and bioactivity

**DOI:** 10.1007/s13205-016-0398-6

**Published:** 2016-02-13

**Authors:** Ushakiranmayi Mangamuri, Vijayalakshmi Muvva, Sudhakar Poda, Krishna Naragani, Rajesh Kumar Munaganti, Bhujangarao Chitturi, Venkateswarlu Yenamandra

**Affiliations:** 1 Department of Botany and Microbiology, Acharya Nagarjuna University, Nagarjunanagar, Guntur, 522510 Andhra Pradesh India; 2 Department of Biotechnology, Acharya Nagarjuna University, Guntur, 522510 Andhra Pradesh India; 3 Organic Chemistry Division-I, Indian Institute of Chemical Technology, Hyderabad, 500007 India

**Keywords:** Mangrove ecosystem, *Streptomyces cheonanensis*, Bioactive compounds, Antimicrobial activity, Cytotoxicity

## Abstract

The strain VUK-A was isolated from a sediment sample of the Coringa mangrove ecosystem was identified as *Streptomyces cheonanensis* based on morphological, physiological, biochemical and molecular properties. Chemical investigation of the secondary metabolites of the strain *Streptomyces cheonanensis* VUK-A has led to the segregation of two bioactive compounds, namely 2-Methyl butyl propyl phthalate (**1**) and Diethyl phthalate (**2**) using column chromatography. The chemical structure of the active compounds was established on the basis of spectroscopic analysis, including ^1^H NMR and ^13^C NMR spectroscopies, FTIR and EIMS. The antimicrobial activity of the bioactive compounds produced by the strain was tested against a wide variety of bacteria and fungi and expressed in terms of minimum inhibitory concentration. The compounds (**1**&**2**) were active against all the bacteria tested, and the best activity of compound **1** was recorded against *Proteus vulgaris* (4 µg/ml). Compounds (**1**&**2**) were active against dermatophytes and fungi but compound **1** displayed high antifungal activity against *Candida albicans* (8 µg/ml) and *Fusarium solani* (16 µg/ml) compared to standard antifungal agents. The cytotoxicity of the bioactive compound **1** was tested against MDA-MB-231, OAW-42, HeLa, and MCF-7 cell lines. The highest activity of 100 µM by compound **1** was recorded against HeLa cancer cell lines. In fact, this is the first report of 2-Methyl butyl propyl phthalate from the genus *Streptomyces*.

**Electronic supplementary material:**

The online version of this article (doi:10.1007/s13205-016-0398-6) contains supplementary material, which is available to authorized users.

## Introduction

Microbes are the factors for a multitude of human diseases, but also equally owe a great extent to human medicine, disease treatment and control. This is due to the fact that marine microbes yield natural products and their synthetic analogs represent a large dimension of the drugs with clinical application and treatment for infectious disease as antibiotics (Jerry Reen et al. 2015). These natural product drugs are often highly potent, active and highly specific to the cellular targets (Koehn and Carter 2005). Introduction of these compounds into clinical practice begins to elevate the antibiotic resistant bacteria with many strains spread across the globe (Woodford et al. 2011; So et al. 2011) due to increased antibiotic usage. The situation is worsened with the promotion of few specific antibiotics in recent years and also decreases of funds for the discovery of the new drugs by the big pharma (So et al. 2011). Most of the recent new drugs derived from the pre-existing scaffolds are active against gram-positive pathogens, but the major threat comes from the gram-negative pathogens where these drugs remain ineffective (Freire-Moran et al. 2011). This validates that drug resistant pathogens coupled with decline in the development of new chemical entities pull the world back into pre antibiotic era unless the situation is decisively addressed forthwith (Pidot et al. 2014). This situation can be improved by not only in the form of discovering new antibiotics but also isolation and identification of new sources of bioactive compounds. The recorded sources for novel drug leads include actinomycetes predominately the genus *Streptomyces* (Watve et al. 2001).

Actinomycetes of mangrove origin remain as an unexploited and luxuriant source of pharmaceutical thrust. The expedition for the microbial consortia with therapeutic properties continues to receive great attention as researchers investigate mangrove microbes for an inclusive stretch of new molecules with antimicrobial and antitumor biochemical activities. Probability studies revealed that the micro biota associated with mangrove ecosystem is more promising when compared to the terrestrial microbes. Mangrove actinobacteria are the rich source for the potent novel secondary metabolites and majority of these compounds are derived from a single genus *Streptomyces* whose species are distributed widely. *Streptomyces,* a gram-positive actinobacteria that produce many pharmaceutically important secondary metabolites include therapeutic enzymes, antibiotics, antitumor agents, vitamins, and immunosuppressant’s (Watve et al. 2001). In fact *Streptomyces* alone accounts for 80 % of the action natural products reported till date whose biosynthetic capacities remain rival to researchers (Panchanathan et al. 2014). Commercial importance is attributed to these strains due to their exclusive capacity to produce potent bioactive metabolites with potent biological activities. The bioactive secondary metabolites from the actinomycetes are endowed with novel chemical skeletons with strong biological activities. Uncaged mangrove habitats provide more chances for isolating new species of *Streptomyces* with unique chemical structures repelling many microbial diseases and cancers (Jose and Jebakumar 2014). Mangrove ecosystems are credited to be a treasure for promising extraordinary metabolites due to the continuous environmental fluctuations such as salinity and tidal forces. This un-trapped microbial diversity of the mangrove sediments is a potential resource for exploring novel bioactive compounds. India has many such unexplored mangrove regions with rich source of novel metabolites (Ganesan et al. 2014). In view of the significance of mangrove ecosystem the present study is aimed to evaluate bio prospective study of bioactive compounds isolated from actinomycetes of mangrove sediment samples together with the extraction, isolation, and structure elucidation of bioactive compounds.

Screening of sediment samples from Coringa mangrove ecosystem for potent actinomycetes led to the isolation of morphologically distinct actinobacterial isolate designated as *Streptomyces cheonanensis* VUK-A by using conventional and molecular methods. A combination of various separation techniques such as solvent extraction, chemical precipitation and repeated column chromatography are employed for the isolation of active compounds from *Streptomyces* *cheonanensis* VUK-A along with the biological evaluation of the pure compounds.

## Materials and methods

### Polyphasic taxonomy

The sediment samples were collected in bimonthly intervals from April 2010 to March 2011 from different locations of mangrove ecosystem of Coringa (Lat. 16°44 to16°53′N; Long. 82°14′ to 82°22′E) situated near the exit of Bay of Bengal along the south east coast of Andhra Pradesh, India. Samples were collected from 6 to 10 cm depth and transported to the laboratory in sterile bags and later air dried at room temperature. The samples taken were analyzed for abiotic parameters such as pH, temperature, humidity and salinity at Department of Soil Science, Acharya NG Ranga Agriculture College, Bapatla. The air dried sediment sample was pre-treated with calcium carbonate (10:1 w/w) and incubated at 37 °C for 4 days. The treated sample was suspended in sterile distilled water (1 g in 100 ml), homogenized by vortexing and 0.1 ml of serially diluted sample (10^−4^ dilution) was spread over the surface of starch casein agar medium containing 3 % NaCl supplemented with nalidixic acid (25 µg/ml) and secnidazole (25 µg/ml). After incubation for a week at 30 °C, distinct strain was selected and maintained by sub culturing on yeast extract-malt extract dextrose (YMD) agar medium at 4 °C for further study. The isolate was preliminarily characterized as described in the International *Streptomyces* Project (ISP). The cultural characteristics of the strain were studied on different media (Shirling and Gottlieb 1966). Micro morphology of the strain was examined by slide culture method (Williams and Cross 1971). Physiological characterization such as the effect of pH, temperature and salinity tolerance was analyzed (Ellaiah et al. 2005). Biochemical tests of the strain were also evaluated (Cowan 1974; Gordon 1966; Jones 1949; Waksman 1961a). Carbohydrate utilization was determined by growing the strain on carbon utilization medium (ISP-9) (Waksman 1961b). Molecular genomic identification of the strain was carried out according to the procedure of Nilsson and Strom (Nilsson and Strom 2002).

### Purification and structural elucidation of bioactive metabolites

The seed broth was prepared by culturing *Streptomyces cheonanensis* VUK-A on ISP-2 medium and incubated on rotary shaker (250 rpm) at 35 °C for 48 h. After 48 h incubation, the seed culture at the rate of 10 % was transferred to the optimized fermentation medium consisting of lactose (1 %), peptone (0.5 %), K_2_HPO_4_ (0.05 %), FeSO_4_ (0.001 %), and NaCl (3 %) with pH adjusted to 7.0 (Ushakiranmayi et al. 2012). The culture filtrates (40 L) obtained after cultivation of the strain for 96 h were extracted twice with ethyl acetate and concentrated in a rotavap, and freeze dried to yield a dark green crude residue. The weight of total crude extract was 2.5 g. The dark green crude residue (2.5 g) was loaded on a silica gel column (25 × 5 cm, Silica gel 100, Merck, Mumbai, India) and eluted successively with 200 ml of 100 % hexane, 200 ml of linear gradient hexane: ethyl acetate (v/v, 75:25−25:75), 200 ml of 100 % ethyl acetate and finally with 200 ml of 100 % methanol, resulting in six fractions, 5 polar residues and one non-polar residue. Among the five polar fractions, two fractions (fraction III & IV) and the single non-polar fraction exhibited high antimicrobial activity. The two polar fractions were rechromatographed using different gradient eluent systems to obtain the fractions in pure form for structural elucidation. Based on the ^1^H-NMR spectral data, the fraction III (900 mg) was selected for further studies and subjected to silica gel column chromatography (100–200 mesh), which afforded fractions 1–4. Based on TLC monitoring and NMR spectral data, the sub fractions 2 and 4 were selected for further purification. The active sub fractions 2 (320 mg) and 4 (240 mg) were subjected to further purification by silica gel column chromatography by using 30 and 40 % ethyl acetate and yielded compound **1** (22 mg) and **2** (34 mg), respectively. The purity of the compounds was checked using TLC and structures of the active compounds were elucidated and confirmed on the basis of FTIR, mass, NMR spectroscopic data.

### Test micro-organisms

Gram-positive bacteria: *Bacillus cereus* (MTCC 430), *Streptococcus mutans* (MTCC 497)*, Staphylococcus aureus* (MTCC 3160), *Staphylococcus epidermis* (MTCC 120), *Bacillus subtilis* (ATCC 6633), *Bacillus megaterium* (NCIM 2187); Gram-negative bacteria: *Escherichia coli* (ATCC 35218), *Pseudomonas aeruginosa* (ATCC 9027), *Proteus vulgaris* (MTCC 7299), *Serratia marcescens* (MTCC 118) *Xanthomonas campestris* (MTCC 2286), *Xanthomonas malvacearum* (NCIM 2954) and *Salmonella typhi* (ATCC 14028); Medically important dermatophytes: *Candida albicans* (ATCC 10231) and *Epidermophyton floccosum* (MTCC 145); Medically and agriculturally important filamentous fungi: *Aspergillus niger* (ATCC 1015), *Aspergillus flavus* (ATCC 9643), *Fusarium oxysporum* (MTCC 3075), *Fusarium solani* (MTCC 4634), *Penicillum citrinum* (MTCC 6489), *Verticillium alboatrum* and *Alternaria alternata* (MTCC 6572). The test micro organisms used in the present study were procured from ATCC, University Boulevard, Manassas, USA and MTCC, Chandigarh, NCIM, Pune, India and preserved at 4 °C.

### Minimum inhibitory concentration (MIC) assay

The antimicrobial spectra of the bioactive compounds of the strain were determined in terms of minimum inhibitory concentration (MIC) against a wide variety of gram-positive and gram-negative bacteria and fungi by using the agar plate diffusion assay (Cappuccino 2002). Triplicate sets of the plates were maintained for each concentration of the test sample. Muller-Hinton agar and Czapek-Dox agar media were prepared to grow the bacteria and fungi, respectively. The purified compounds were dissolved in dimethyl sulfoxide at concentrations ranging from 0 to 1000 μg/ml and used to assay against supra mentioned test bacteria and fungi. The inoculated plates were examined after 24–48 h of incubation at 37 °C for bacteria and 48–72 h at 28 °C for fungi. The lowest concentration of the bioactive metabolites exhibiting significant antimicrobial activity against the test microbes was taken as the MIC of the compound.

### Cell proliferation (MTT) assay

The Cytotoxicity of the compound **1** was assessed on the basis of the measurement of the in vitro growth in the 96-well plates by cell mediated reduction of tetrazolium salt to water insoluble formazan crystals, as per the micro culture MTT assay (Mosmann 1983). Cell lines for testing in vitro cytotoxicity included human breast adenocarcinoma cell line (MDA-MB-231), human cervical cancer cell line (HeLa), human ovarian cyst adenocarcinoma cell line (OAW-42) and human breast adenocarcinoma cell line (MCF-7) (cell lines reported to be resistant to cancer drugs) obtained from National Centre for Cell Science, Pune, India. Cell lines MDA-MB-231, HeLa and OAW-42 were cultured on Dulbecco’s modified Eagle’s medium supplemented with fetal bovine serum (10 %; v/v), l-glutamine (2 mM), penicillin (10 units/ml) and streptomycin (10 μg/ml), while Breast cancer cell line MCF-7 was cultured on Roswell Park Memorial Institute medium 1640 supplemented with fetal bovine serum (10 %; v/v), l-glutamine (2 mM), penicillin (10 units/ml), and streptomycin (10 μg/ml), all in a humidified atmosphere (95 %) with 5 % of CO_2_ at 37 °C. Cells were seeded in 96-well micro titer plates at a density of 5 × 10^3^ per well (100 µl) containing 0.1 ml of medium. After overnight incubation, the cells were treated with different test concentrations of bioactive compounds (10, 100, 1000, and 5000 µM) at identical conditions with three replicates of each concentration. After 24 h of incubation, the cell viability was assessed by adding 20 µl of MTT (5 mg/ml in PBS) per well and the plates were incubated at 37 °C for 4 h. The formazan crystals formed in the cells were dissolved with 100 µl of 0.1 % acidified isopropanol, and the rate of color development was measured at 570 nm using a micro plate reader. The IC_50_ value (50 % inhibitory concentration) of the compound was calculated using Sigma Plot software with reference to that of Taxol at 10 ηM as standard. All the experiments were carried out in triplicates.

## Results and discussion

### Polyphasic taxonomy

The seasonal variations of different abiotic parameters investigated were as follows: pH (6.4–8.59), temperature (18.6–39.4 °C), humidity (35.4–91.8 %) and salinity (10.55–38.21 psu). Marked fluctuations were noticed in pH values in mangrove ecosystem of Coringa with low value recorded during monsoon (6.4 in June) but the highest value (8.59) was recorded during December. Atmospheric average temperature in the present study ranged between 18.6 and 39.4 °C with maximum during summer while minimum during monsoon. The relative humidity ranged between 35.4 % in summer and 91.8 % during monsoon with an annual average of 61 %. The highest salinity values were observed in the summer season (38.21 psu) and minimum in pre-monsoon (10.55 psu).

Screening of soil samples from Coringa mangrove ecosystem for potent actinomycetes led to the isolation of morphologically distinct actinomycete isolate VUK-A. The strain exhibited good growth on ISP-1, ISP-2, ISP-7, starch casein agar, Czapek-Dox agar and maltose tryptone agar, moderate on ISP-4, ISP-5 and nutrient agar, while growth was poor on ISP-3 agar media. Morphological and micro morphological observations of the strain revealed extensively branched mycelium and bear short chain of spores. The color of aerial mycelium varied from grey to white while the substrate mycelium varied from dark brown to black. Green pigment production by the strain was found on the culture media while melanin pigmentation was found on tyrosine (ISP-7) agar media. Sporophore morphology of the strain was examined through scanning electron microscopy and it was grouped under rectus-flexibilis type of *Streptomyces*. Growth of the strain occurred in the pH range of 6–9 with an optimum growth at pH 7. The temperature range for growth was 25–45 °C with the optimum temperature being 30 °C. The strain exhibited salt tolerance up to 7 % with optimum growth at 3 % NaCl. VUK-A exhibited positive response to catalase production and citrate utilization but negative for indole, methyl red, vogues-proskauer, urease, hydrogen sulfide production, and nitrate reduction tests. The strain efficiently utilized lactose, d-glucose, maltose, sucrose, galactose, fructose, starch, and arabinose as carbon sources but not xylose and mannitol. 16S rDNA gene sequence of the isolate VUK-A showed a close relation with *Streptomyces cheonanensis.* The rDNA sequence was deposited in the NCBI Gen Bank with an accession number JN087502 (Ushakiranmayi et al. 2011).

The crude extract was subjected to silica gel column chromatography using a gradient solvent system of hexane: ethyl acetate. Among the six fractions collected the fraction III and IV exhibiting good antimicrobial activity was rechromatographed on a silica gel column and yielded compounds **1** and **2,** respectively.

**Fig.1:**
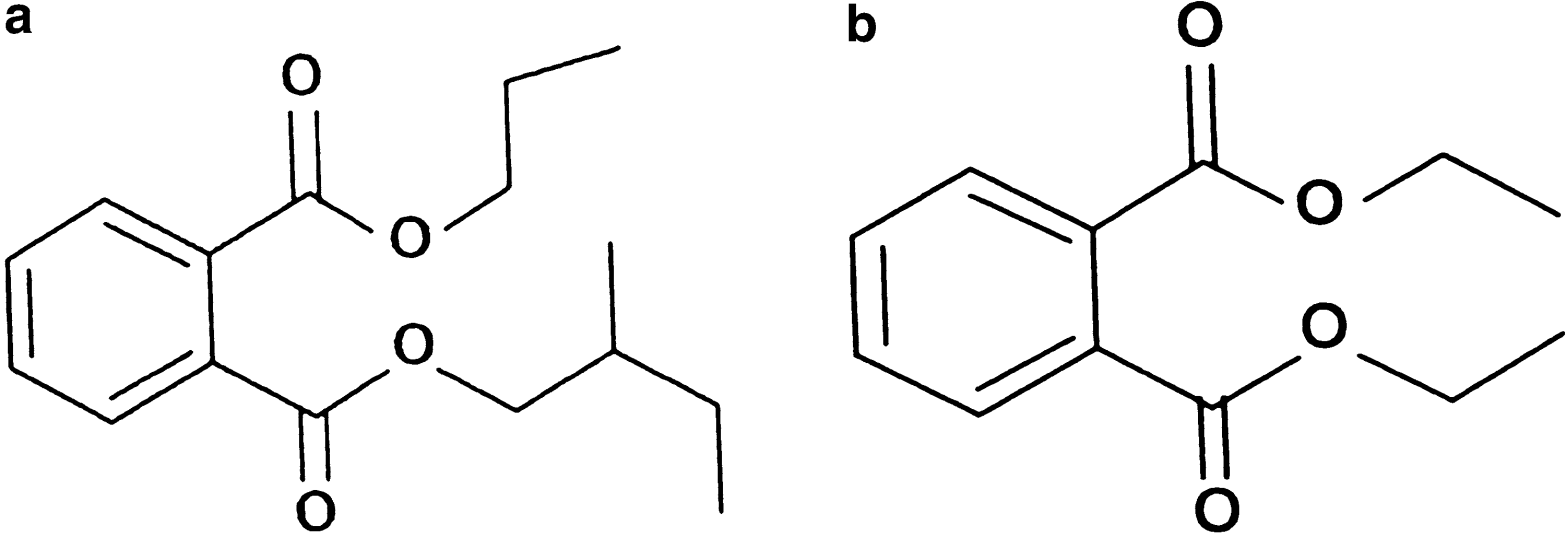
Molecular structures of **a** 2-Methyl butyl propyl phthalate (**1**) **b** Diethyl phthalate (**2**)

Compound **1** was obtained as a white solid, soluble in dimethylsulfoxide, methanol, ethanol, and chloroform. The ^1^H-NMR spectrum of the compound **1** showed 9 signals at 7.71 (m, 2H); 7.52 (m, 2H); 4.30 (*t*, 2H, *J* = 6.46 Hz); 4.07 (d, 2H, *J* = 7.27 Hz); 2.04 (m, 1H); 1.71 (*t*, 3H, *J* = 7.27 Hz); 1.44 (m, 2H); 0.98 (m, 6H); and 0.86 (m, 3H) (Supplementary Fig. A); while ^13^C exhibited 11 signals at 167.6, 132.2, 130.8 (2C), 128.0 (2C), 71.7, 65.5, 30.5, 29.6, 27.6, 19.1, and 13.7 (Supplementary Fig. B). EIMS analysis of the compound gave a molecular ion m/z at 300 [M + Na]^+^ (Supplementary Fig. C). The IR spectrum exhibited absorption bands at V_max_ 1726 cm^−1^ indicating C=O group in the structure (Supplementary Fig. D). Based on the above spectral data, bioactive compound **1** was identified as 2-Methyl butyl propyl phthalate (Fig. 1a) with the molecular formula of C_16_H_22_O_4_.

Compound **2** was obtained as a colorless, oily liquid soluble in ether, methanol, ethanol and chloroform. The ^1^H-NMR spectrum of the compound **2** showed signals at δ 7.27 (m, 2H); 7.52 (m, 2H); 4.235 (q, 4H, *J* = 6.78; 4.35 Hz) and 1.37 (t, 6H, *J* = 7.55 Hz) (Supplementary Fig. E), while ^13^C exhibited 6 signals at δ 167.6 (2C); 132.1 (2C); 130.8 (2C); 128.7 (2C); 61.5 (2C); and 14.0 (2C) (Supplementary Fig. F). EIMS analysis of the compound gave a molecular ion m/z at 245 [M + Na]^+^ (Supplementary Fig. G). The IR spectrum displayed absorption bands at V_max_ 1727 cm^−1^ indicating C = O group in the structure (Supplementary Fig. H). Based on the above spectral data, bioactive compound **2** was identified as Diethyl phthalate (Fig. 1b) with the molecular formula of C_12_H_14_O_4_.

### Minimum inhibitory concentration (MIC) assay

Antibacterial activities of the bioactive compounds (**1** and **2)** in terms of MIC are shown in Table 1. The bioactive compounds exhibited antibacterial activity against a variety of gram-positive and gram-negative bacteria, for which the MIC values ranged from 4 to 256 μg/ml. Among the facultative and pathogenic gram-positive bacteria, compound **1** was active against all the bacteria tested and the best activity of this compound was recorded against *Staphylococcus aureus* (8 μg/ml) followed by *Streptococcus mutans* and *Bacillus subtilis* (16 μg/ml). Compound **2** presented highest activity against *Staphylococcus epidermis* (16 μg/ml) followed by *S. mutans* (32 μg/ml). Of the gram-negative bacteria, the microorganisms that presented highest sensitivity towards compound **1** was *Proteus vulgaris* (4 μg/ml) followed by *Escherichia coli* (16 μg/ml). Compound **2** recorded highest activity against *E. coli* (32 μg/ml) followed by *Pseudomonas aeruginosa* and *Xanthomonas malvacearum* (64 μg/ml). Tetracycline served as reference control for antibacterial activity. Compared with the standard drug tetracycline, compound **1** displayed high sensitivity against *P. vulgaris*, *S. aureus*, *S. mutans* and *Bacillus subtilis* and recorded similar sensitivity like positive control against *Serratia marcescens*, while compound **2** displayed similar sensitivity like positive control against *S. mutans* and *Staphylococcus epidermis* (Table 1). Tetracycline, in other cases, showed good antibacterial activity over the metabolites of the strain.

**Table 1:** Minimum inhibitory concentration (μg/ml) of the bioactive compounds produced by *Streptomyces cheonanensis* VUK-A against gram-positive and gram-negative bacteria

Test microorganisms	Compound-**1**	Compound-**2**	Tetracycline
*S. aureus*	8 ± 0.03	64 ± 0.01	32 ± 0.03
*S. mutans*	16 ± 0.01	32 ± 0.00	32 ± 0.03
*S. epidermis*	64 ± 0.02	16 ± 0.00	16 ± 0.03
*X. campestris*	32 ± 0.01	128 ± 0.01	16 ± 0.03
*X. malvacearum*	64 ± 0.04	64 ± 0.02	8 ± 0.03
*B. subtilis*	16 ± 0.01	128 ± 0.02	32 ± 0.03
*B. megaterium*	64 ± 0.00	64 ± 0.01	16 ± 0.03
*B. cereus*	32 ± 0.02	64 ± 0.03	8 ± 0.03
*E. coli*	16 ± 0.00	32 ± 0.03	8 ± 0.03
*P. aeruginosa*	64 ± 0.01	64 ± 0.00	8 ± 0.03
*S. marcescens*	32 ± 0.01	128 ± 0.02	32 ± 0.03
*P. vulgaris*	4 ± 0.02	128 ± 0.01	16 ± 0.03
*S. typhi*	128 ± 0.03	256 ± 0.01	8 ± 0.03

Values are mean ± standard deviation (*n* = 3)

Compound-**1**: 2-Methyl butyl propyl phthalate

Compound-**2**: Diethyl phthalate

Antibiotic: Tetracycline

Antifungal activity against dermatophytes and filamentous fungi and the corresponding MIC values are recorded in Table 2. Compound **1** exhibited significant MIC value against *Candida albicans* (8 μg/ml) whereas compound **2** recorded sensitivity of 32 μg/ml against supra said dermatophyte. Among the filamentous fungi tested, *Fusarium solani* recorded sensitivity of 16 μg/ml followed by *Fusarium oxysporum* and *Aspergillus niger* (32 μg/ml) towards compound **1**. Compound **2** was active against *Aspergillus flavus* at 32 μg/ml, and for this compound *Alternaria alternata* recorded no activity up to 512 μg/ml. Compared to standard drugs griseofulvin and amphotericin-B compound **1** displayed high sensitivity against *Candida albicans* and *Fusarium solani* while Compound **2** exhibited lower antifungal activity than the standard fungicides.

**Table 2:** Minimum inhibitory concentration (MIC) of bioactive compounds isolated from *Streptomyces cheonanensis* VUK-A (MIC-(μg/ml)) against dermatophytes and fungi

Dermatophytes	Compound-**1**	Compound-**2**	^*^ Antifungal agent
*C. albicans*	8 ± 0.01	32 ± 0.02	16 ± 0.01
*E. floccosum*	32 ± 0.01	128 ± 0.01	16 ± 0.01
Fungi			
*A. niger*	32 ± 0.00	128 ± 0.01	16 ± 0.02
*A. flavus*	64 ± 0.02	32 ± 0.02	8 ± 0.00
*F. oxysporum*	32 ± 0.03	128 ± 0.02	16 ± 0.00
*F. solani*	16 ± 0.01	64 ± 0.01	32 ± 0.01
*P. citrinum*	64 ± 0.02	256 ± 0.01	8 ± 0.01
*V. alboatrum*	128 ± 0.01	256 ± 0.01	64 ± 0.02
*A. alternata*	128 ± 0.00	>512 ± 0.02	32 ± 0.01

Values are mean ± standard deviation (*n* = 3)

* Antifungal agent: Griseofulvin against dermatophytes and Amphotericin-B against fungi

Compound-**1**: 2-Methyl butyl propyl phthalate

Compound-**2**: Diethyl phthalate

### Cell proliferation (MTT) assay

**Fig. 2:**
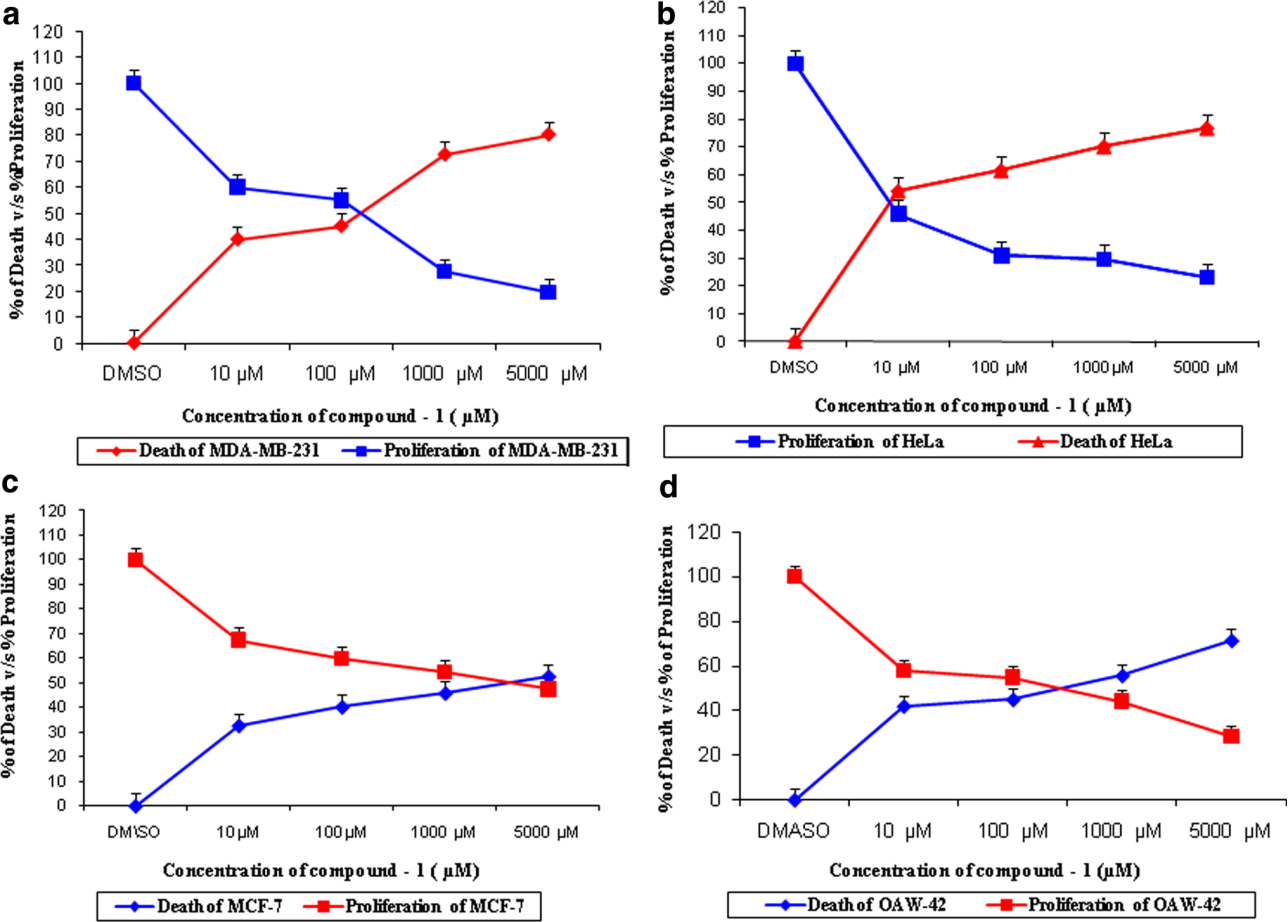
Dose response curve of compound 1 on the growth of **a** MDA-MB-231, **b** HeLa, **c** MCF-7, **d** OAW-42 cancer cell lines

The cytotoxicity of the purified compound **1** was assayed against MDA-MB-231, HeLa, MCF-7 and OAW-42. The results exhibited that compound **1** was active against the four cell lines. The activity of compound **1** against MDA-MB-231, HeLa, MCF-7 and OAW-42 cell lines is presented in Fig. 2a–d. Compound **1** exhibited significant cytotoxicity with MDA-MB-231, HeLa, MCF-7 and OAW-42 cell lines, exhibiting IC_50_ values of 1000 µM (72.5, 55.7 %) (MDA-MB-231) (OAW-42), 100 µM (61.6 %) (HeLa) and 5000 µM (52.6 %) (MCF-7). Taxol, an anti-cancer drug used as the standard, recorded an IC_50_ value of 10 nM (59, 60, 57, and 63 %) against MDA-MB-231, HeLa, OAW-42, and MCF-7 cell lines.

## Discussion

During the course of our screening programme for bioactive secondary metabolites from Coringa mangrove ecosystem, *Streptomyces cheonanensis* VUK-A exhibited significant bioactivity. A novel 2-Methyl butyl propyl phthalate (**1**) along with a known compound Diethyl phthalate (**2**), was isolated from the fermentation broth of the strain grown on lactose-peptone broth. Phthalate compounds are petrochemicals used as plasticizers or solvents in a variety of industrial products and also used in food handling and storage while some of them are considered to be ubiquitous pollutants, with slight endocrine disrupting properties (Cespedes et al. 2004). Phthalate derivatives which are biologically active compounds are also effective against demodicidosis and also drug channeling agents (Makhija and Vavia 2003; Marchetti et al. 2002). Many phthalate derivatives have been isolated from plants, terrestrial and marine microorganisms, fungal and bacterial culture broths, especially from the genus *Streptomyces* (Sastry and Rao 1995; El-Naggar 1997; Chen 2004; Roy et al. 2006). The isolation of compound **2** was previously reported as a natural product from *Streptomyces* sp.1010, isolated from shallow sea sediment from the region of Livingston Island, Antarctica (Ivanova et al. 2001) and *Helicobacter pylori* (Keire et al. 2001). It has been reported that complexes derived from diethyl phthalate have antifungal activity (Raman and Parameswari 2007). Many phthalate derivatives such as bis-(2-ethyl hexyl) phthalate, bis-(5-ethyl heptyl) phthalate and dibutyl phthalates were reported from *Streptomyces* sp.TN 256 strain, *Streptomyces bangladeshiensis* and *Nocardia levis* exhibited several antimicrobial activities (Smaoui et al. 2012; Kavitha et al. 2009). However this is the first report of compound 2-Methyl butyl propyl phthalate (**1**), from the genus *Streptomyces,* and no information is available on the isolation and characterization of 2-Methyl butyl propyl phthalate from microorganisms, particularly actinomycetes.

Our results showed that compound **1** has a good potential inhibitor against *P. vulgaris* (responsible for human urinary tract infections) (4 μg/ml), *S. aureus* (causes impetigo, carbuncles and abscesses) (8 μg/ml), *Candida albicans* (causes oral thrush and vaginal infection) (8 μg/ml) and *F. solani* (responsible for fusarium wilt, fungal keratitis and onychomycosis) (16 μg/ml). Compound **2** showed to have antimicrobial activity against *S. epidermis* (an opportunistic human pathogen responsible for nosocomial infections) (16 μg/ml), *E. coli* (causes cholecystitis, bacteremia, cholangitis and urinary tract infection) (32 μg/ml), *C. albicans* (32 μg/ml) and *Aspergillus flavus* (responsible for pulmonary aspergillosis, production of significant quantities of aflatoxin which is acutely toxic and carcinogenic) with an MIC of 32 μg/ml.

The potential of compound **1** was investigated to inhibit cancer cell growth in MDA-MB-231, HeLa, OAW-42 and MCF-7 cell lines. The compound showed good inhibitory potentiality against supra said cell lines, with remarkable display of activity against HeLa (100 µM). In addition, anti cancer activity of compound **1** against above said cell lines are reported here for the first time. The results of the present study showed that the compound exhibit potent anti cancer activities in impressive low concentrations.

## Conclusion

The two bioactive compounds (**1** & **2**) of the present work extracted from the strain VUK-A exhibited significant antimicrobial activity against opportunistic and pathogenic bacteria and fungi. The compound **1** showed potent cytotoxicity against MDA-MB-231, HeLa, MCF-7 and OAW-42 cell lines. There is no information available on the antimicrobial and cytotoxicity of the reported bioactive compound **1** and this is the first report of isolation and characterization of 2-Methyl butyl propyl phthalate from the genus *Streptomyces.*

## Electronic supplementary material

Below is the link to the electronic supplementary material.

Supplementary material 1 (DOCX 3284 kb)
