# Supplementary material for: Bioactive metabolites produced by Streptomyces Cheonanensis VUK-A from Coringa mangrove sediments: isolation, structure elucidation and bioactivity
Source: 3 Biotech. 2016 Feb 13;6(1):63. doi: 10.1007/s13205-016-0398-6 (PMC4752944; doi:10.1007/s13205-016-0398-6)
Supplement: Supplementary file 1 — Supplementary material 1 (DOCX 3284 kb) [file 13205_2016_398_MOESM1_ESM.docx]

**Supplementary Information**

**Bioactive Metabolites Produced By *Streptomyces Cheonanensis* VUK-A from Coringa Mangrove Sediments: Isolation, Structure Elucidation and Bioactivity**

**Ushakiranmayi Mangamuri^1^, Vijayalakshmi Muvva^1^*, Sudhakar Poda^2^, Krishna Naragani^1^, Rajesh Kumar Munaganti^1^, Bhujangarao Chitturi^4^, Venkateswarlu Yenamandra^4^**

***^1^Department of Botany & Microbiology, Acharya Nagarjuna University, Guntur-522510, Andhra Pradesh, India.***

***^2^Department of Biotechnology, Acharya Nagarjuna University, Guntur-522510, Andhra Pradesh, India.***

***^3^Molecular and Cellular Oncology Laboratory, Department of Biochemistry, School of Life sciences, University of Hyderabad, Hyderabad-500046, India.***

***^4^Organic Chemistry Division-I, Indian institute of Chemical Technology, Hyderabad-500007, India.***

****Corresponding author:***

***Prof. M. Vijayalakshmi***

***Dean, Life Sciences***

***Acharya Nagarjuna University***

***Nagarjunanagar, Guntur-522510***

***Andhra Pradesh, India***

***Email:*** [***profmvl@gmail.com***](mailto:profmvl@gmail.com)

***Phone No: +91-9440870026***

***Fax No: +91-0863-2293378***


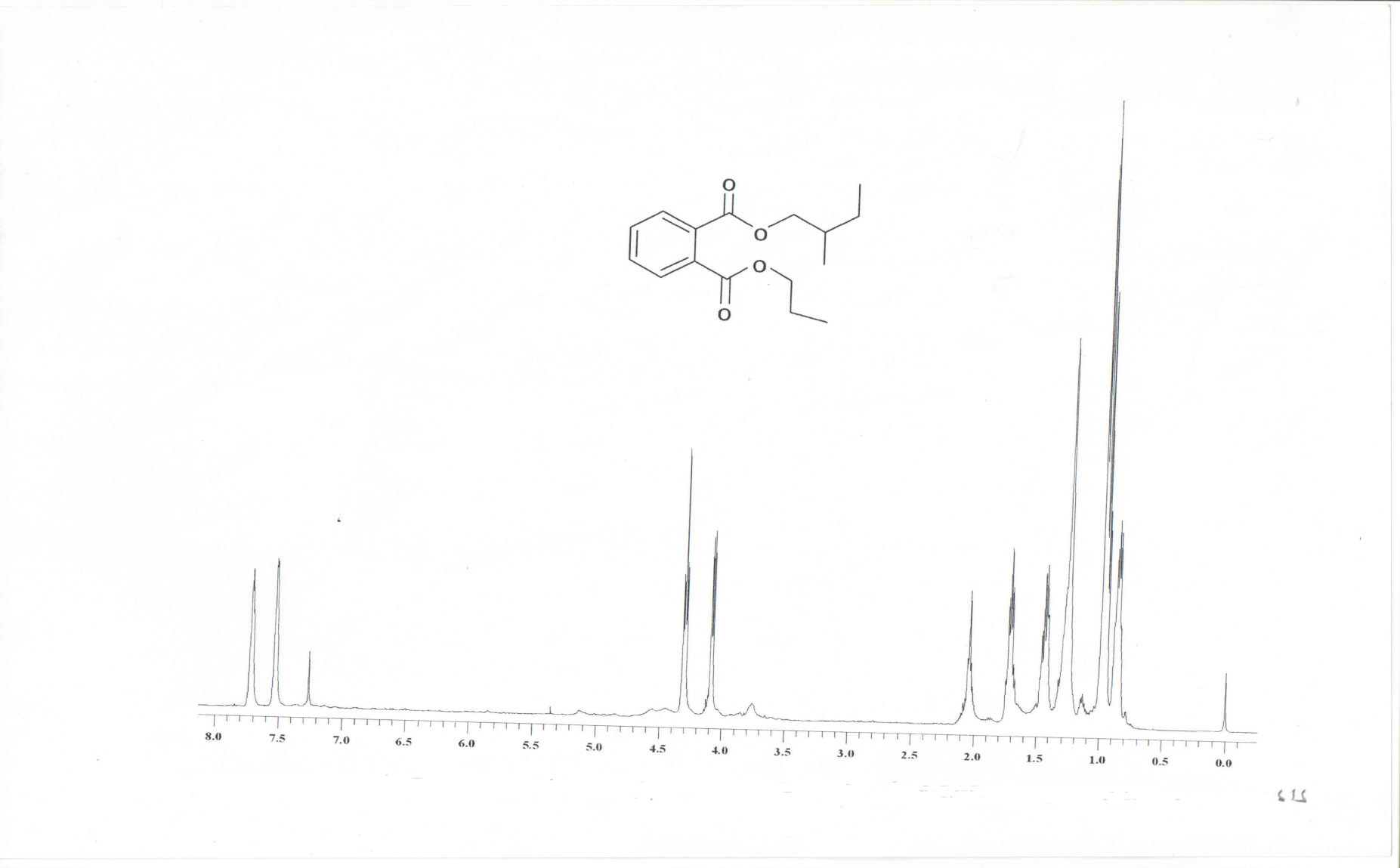


Fig. A ^1^HNMR spectrum of the compound **1** produced by *Streptomyces cheonanesis* VUK-10


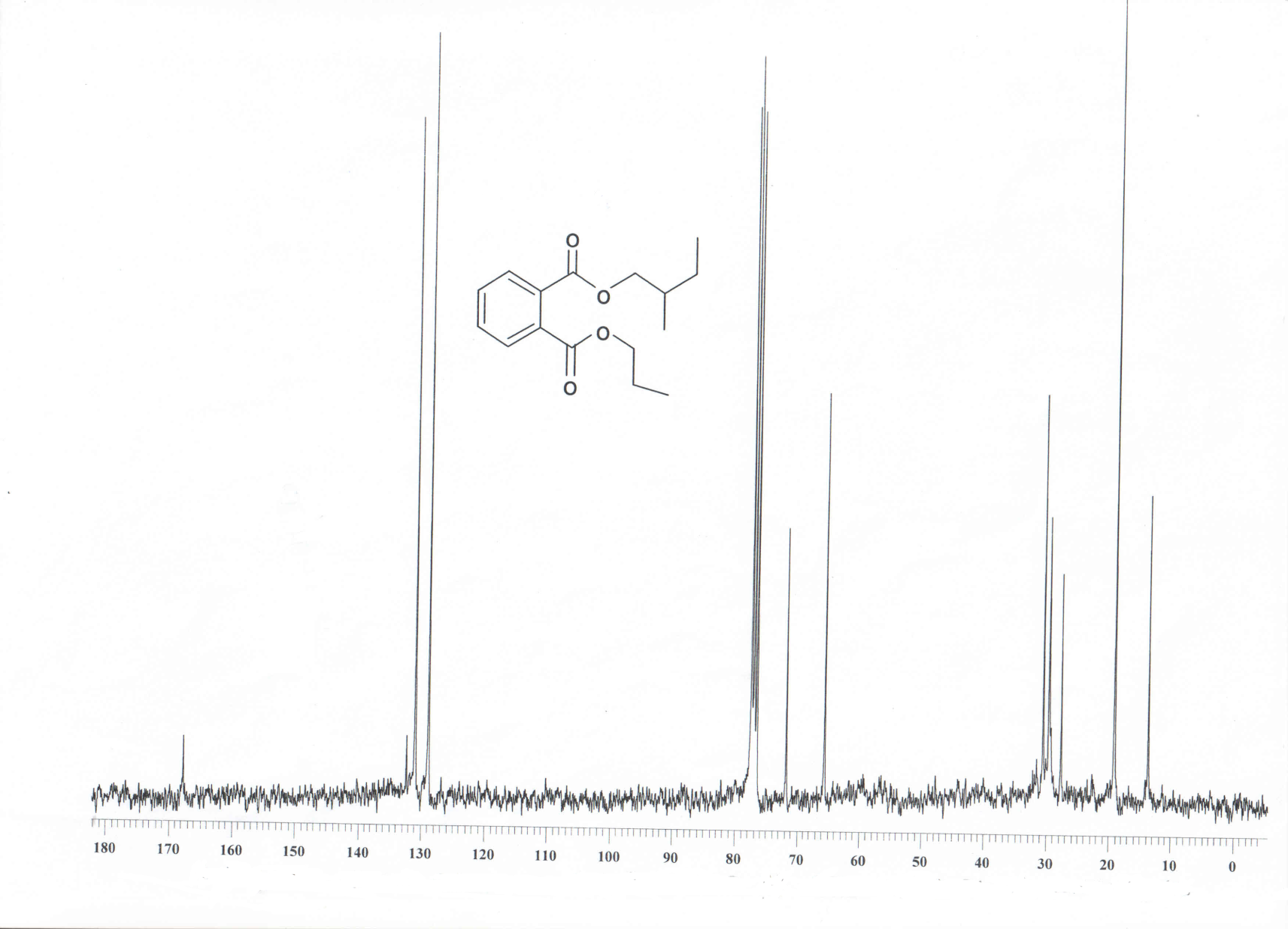


Fig. B ^13^ CNMR of the compound **1** produced by *Streptomyces cheonanesis* VUK-10


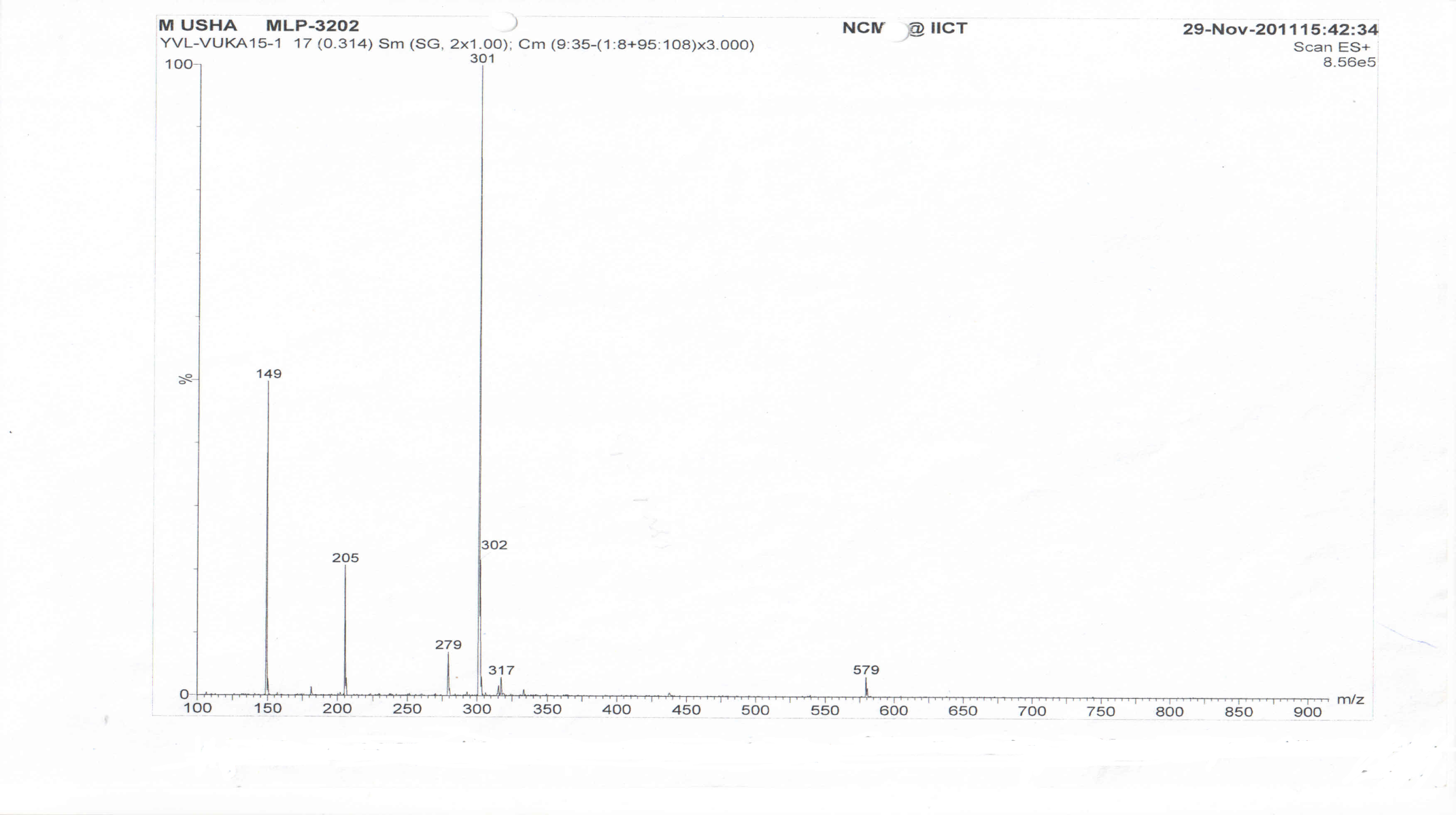


Fig. C Mass spectrum of the compound **1** produced by *Streptomyces cheonanesis* VUK-10


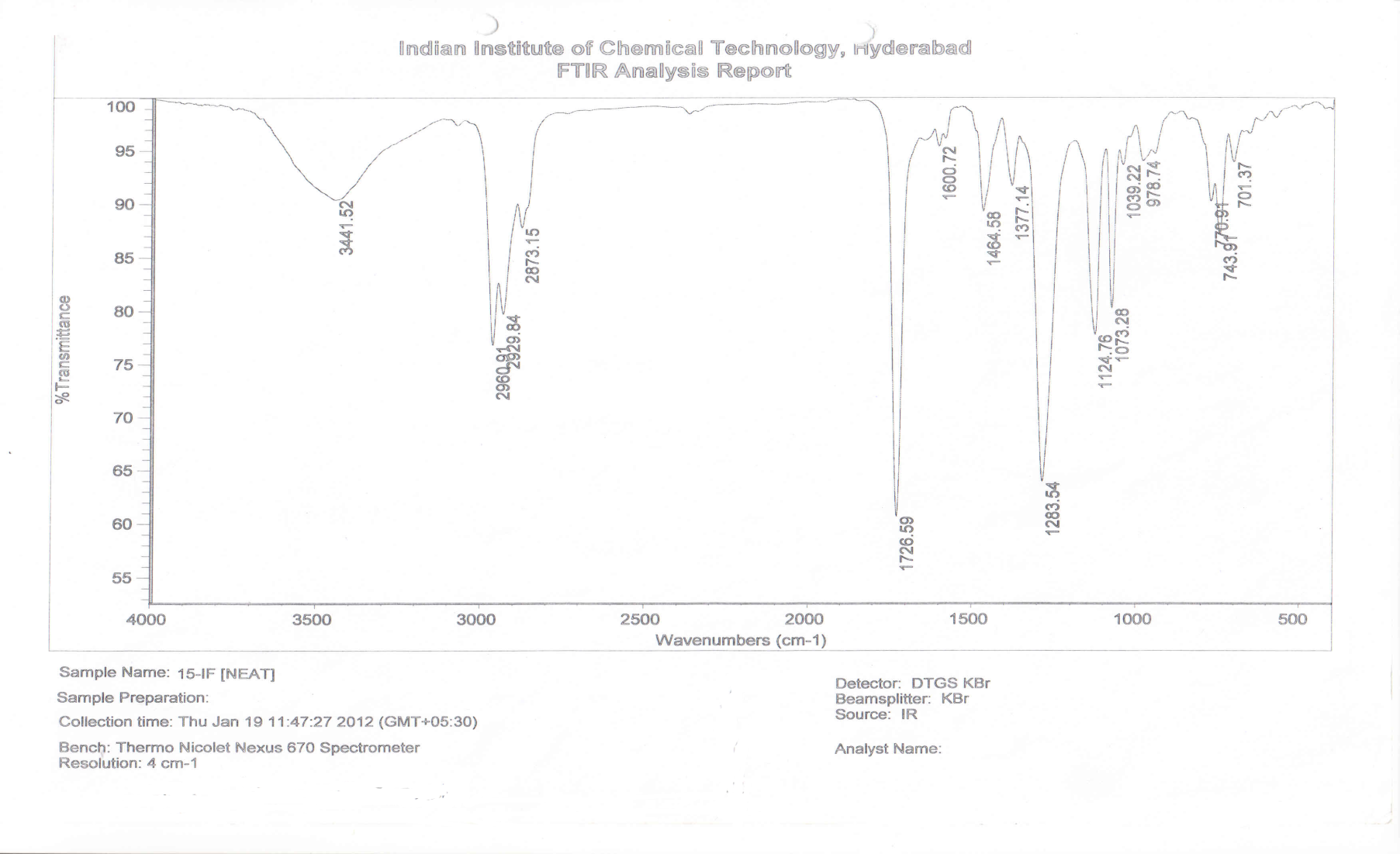


Fig. D FTIR Spectrum of the compound **1** produced by *Streptomyces cheonanesis* VUK-10


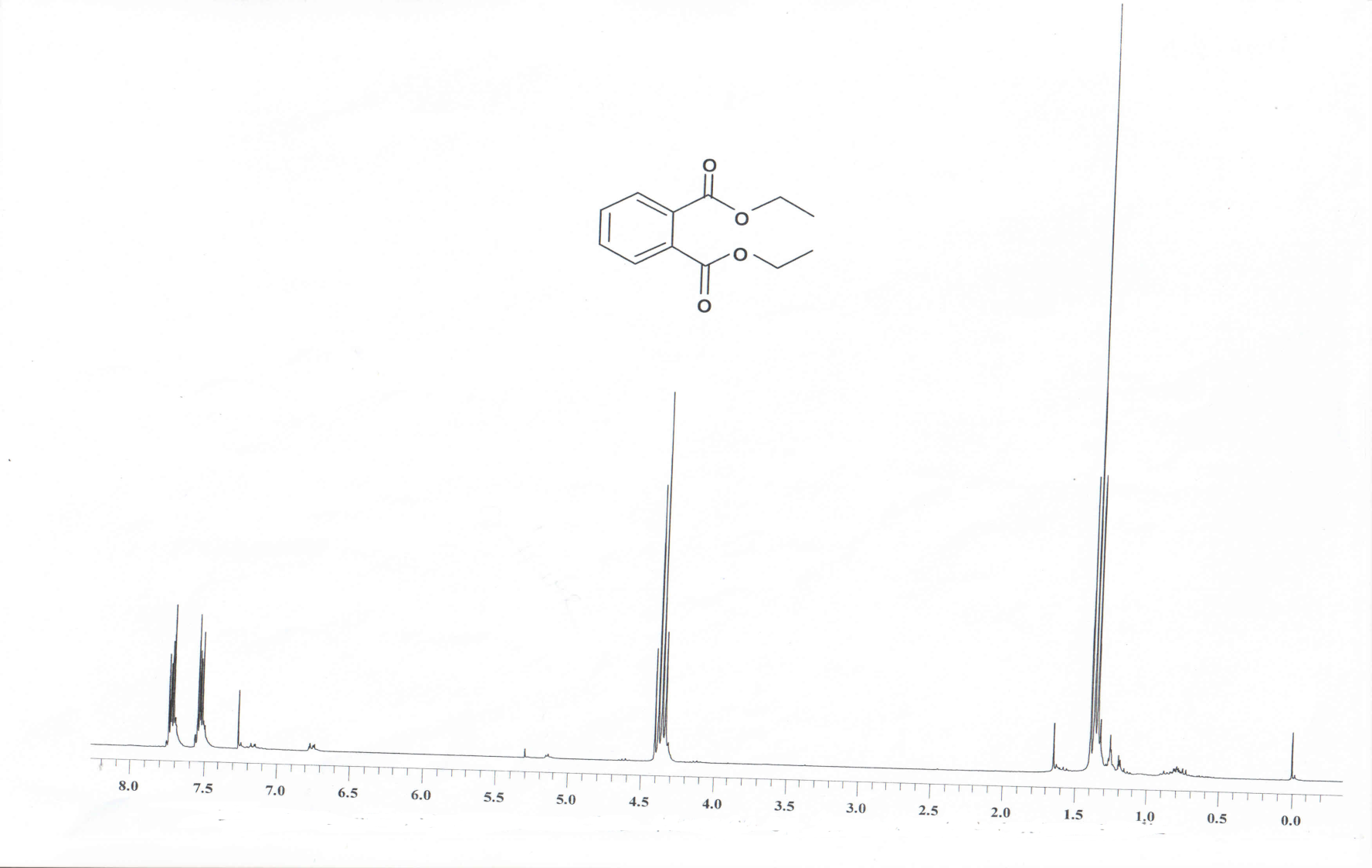


Fig. E ^1^HNMR spectrum of the compound **2** produced by *Streptomyces cheonanesis*


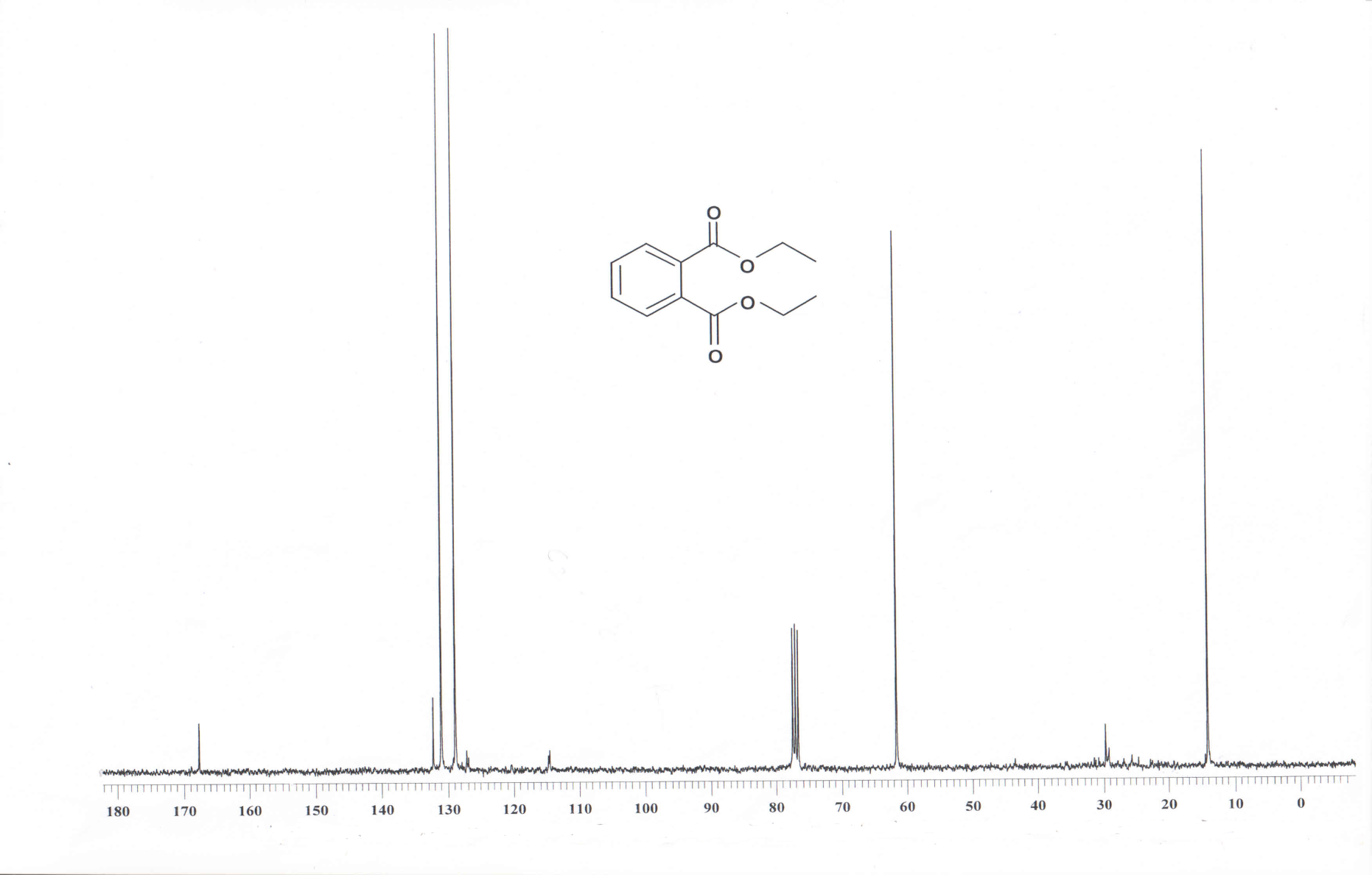


Fig. F ^13^ CNMR spectrum of the compound **2** produced by *Streptomyces cheonanesis*

*
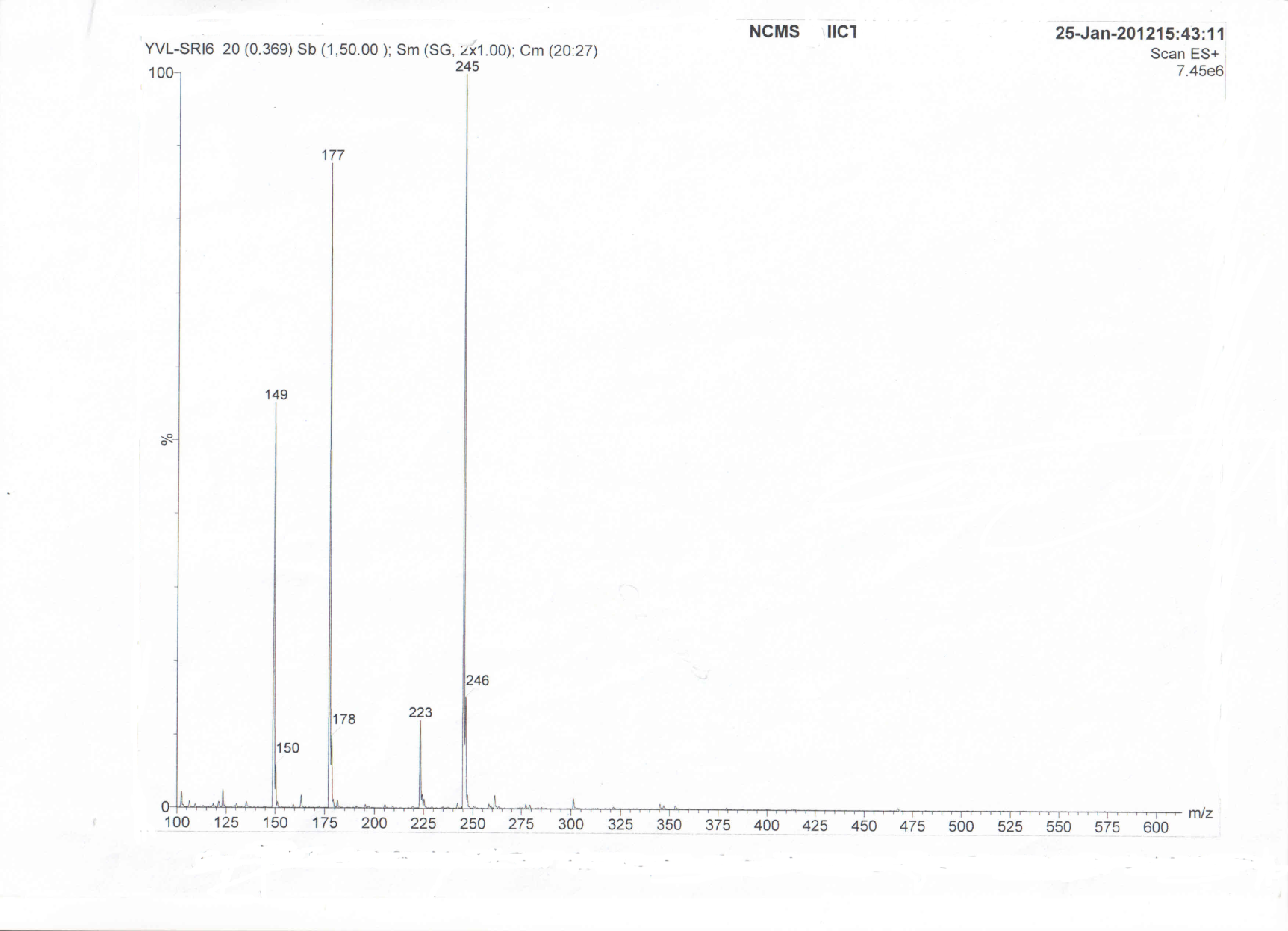
*

Fig. G Mass spectrum of the compound **2** produced by *Streptomyces cheonanesis*


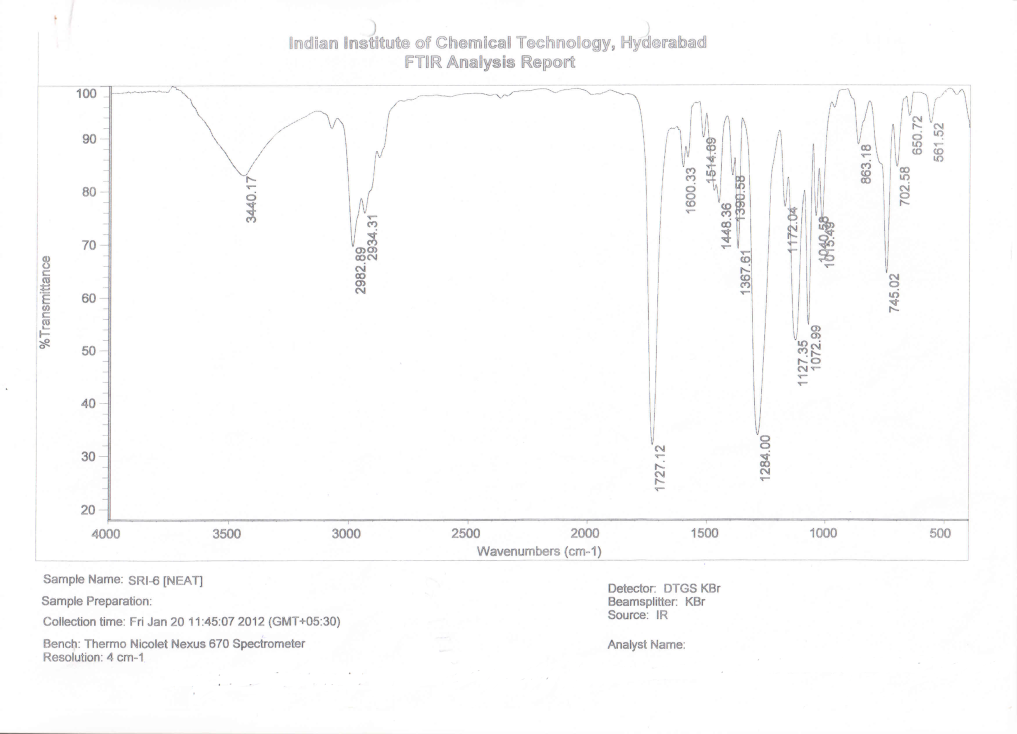


Fig. H FTIR Spectrum of the compound **2** produced by *Streptomyces cheonanensis*
